# Thrombin-Induced Microglia Activation Modulated through Aryl Hydrocarbon Receptors

**DOI:** 10.3390/ijms241411416

**Published:** 2023-07-13

**Authors:** Meei-Ling Sheu, Liang-Yi Pan, Cheng-Ning Yang, Jason Sheehan, Liang-Yu Pan, Weir-Chiang You, Chien-Chia Wang, Hung-Chuan Pan

**Affiliations:** 1 Institute of Biomedical Sciences, National Chung-Hsing University, Taichung 40227, Taiwan; mlsheu@nuchu.edu.tw; 2 Department of Medical Research, Taichung Veterans General Hospital, Taichung 40210, Taiwan; 3 Ph.D. Program in Translational Medicine, Rong Hsing Research Center for Translational Medicine, National Chung Hsing University, Taichung 40227, Taiwan; 4 Faculty of Medicine, Kaohsiung Medical University, Kaohsiung 80708, Taiwan; pan0911606850@gmail.com; 5 Department of Dentistry, School of Dentistry, College of Medicine, National Taiwan University, Taipei 106319, Taiwan; cnyang880@yahoo.com.tw; 6 Department of Neurosurgery, University of Virginia, Charlottesville, VA 22904, USA; jps2f@hscmail.mcc.virginia.edu; 7 Faculty of Medicine, Poznan University of Medical Sciences, 61-701 Poznań, Poland; mattpan9009@gmail.com; 8 Department of Radiation Oncology, Taichung Veterans General Hospital, Taichung 40210, Taiwan; bigjohnyou@gmail.com; 9 Department of Life Sciences, National Central University, Taoyuan 32001, Taiwan; superdukewang@gmail.com; 10 Department of Neurosurgery, Taichung Veterans General Hospital, Taichung 40210, Taiwan

**Keywords:** thrombin, microglia, aryl hydrocarbon receptor, inflammation, neurodegenerative disorder

## Abstract

Thrombin is a multifunctional serine protein which is closely related to neurodegenerative disorders. The Aryl hydrocarbon receptor (AhR) is well expressed in microglia cells involving inflammatory disorders of the brain. However, it remains unclear as to how modulation of AhR expression by thrombin is related to the development of neurodegeneration disorders. In this study, we investigated the role of AhR in the development of thrombin-induced neurodegenerative processes, especially those concerning microglia. The primary culture of either wild type or AhR deleted microglia, as well as BV-2 cell lines, was used for an in vitro study. Hippocampal slice culture and animals with either wild type or with AhR deleted were used for the ex vivo and in vivo studies. Simulations of ligand protein docking showed a strong integration between the thrombin and AhR. In thrombin-triggered microglia cells, deleting AhR escalated both the NO release and iNOS expression. Such effects were abolished by the administration of the AhR agonist. In thrombin-activated microglia cells, downregulating AhR increased the following: vascular permeability, pro-inflammatory genetic expression, MMP-9 activity, and the ratio of M1/M2 phenotype. In the in vivo study, thrombin induced the activation of microglia and their volume, thereby contributing to the deterioration of neurobehavior. Deleting AhR furthermore aggravated the response in terms of impaired neurobehavior, increasing brain edema, aggregating microglia, and increasing neuronal death. In conclusion, thrombin caused the activation of microglia through increased vessel permeability, expression of inflammatory response, and phenotype of M1 microglia, as well the MMP activity. Deleting AhR augmented the above detrimental effects. These findings indicate that the modulation of AhR is essential for the regulation of thrombin-induced brain damages and that the AhR agonist may harbor the potentially therapeutic effect in thrombin-induced neurodegenerative disorder.

## 1. Introduction

Neuroinflammation has been well known for decades to be involved in the process of neurodegenerative diseases. However, it is still not fully understood when and how inflammation arises in the development of neurodegenerative disorder [1]. Whether inflammation is a trigger factor or part of the cascade after onset of the neurodegenerative process remains unclear. Mounting evidence supports that microglial activation is a major contributor to neuronal damage in neurodegenerative disorder [2,3,4]. Microglia, the tissue macrophages of the brain, in healthy conditions, displays a resting phenotype that is characterized by a ramified morphology with fine processes that are well suited for constantly scanning their environment. Upon homeostatic disturbances, microglia rapidly change phenotypes for tissue remodeling and neurogenesis following pathological processes such as inflammation, stroke, and trauma [4,5,6].

Thrombin is prominent in its function as the serine protease in the coagulation cascade [7]. Thrombin is, in general, a potent signaling molecule in regulating both physiologic and pathogenic responses in various cells [1,4,6,8]. Accompanying CNS injury or cerebral vascular damages, prothrombin activation and leakage of active thrombin into CNS parenchyma leads to over-reactive inflammatory responses, often causing irreversible neuronal damages. Thrombin not only activates endothelial cells and induces leukocyte infiltration and edema, but it also activates microglia to propagate the focal inflammation, producing neurotoxic effects [4,6,8].

Aryl hydrocarbon receptor (AhR) is a member of the PAS (Per-ARNT-Sim) family. It has a basic domain of helix-loop-helix, and it appears in the cytoplasm of many vertebrate cells as part of a complex. This complex has a dimer of the chaperone heat shock protein (HSP) 90, an immunophilin-like protein called X-associated protein (XAP) 2 or AIP1, the phosphoprotein p23, and the non-receptor protein tyrosine kinase (known as pp60src) [9]. AhR regulates neural functions such as xenobiotics and through AhR ligand, 2,3,7,8-tetrachlorodibenzo-p-dioxin (TCDD), controls neuronal proliferation, differentiation, and survival [10]. AhR can be visualized in animal models using immunohistochemical stain or in situ hybridization [11]. AhR messenger RNA (mRNA) is expressed in a spatiotemporal fashion in multiple brain areas such as the cerebral cortex, hippocampus, cerebellum, olfactory bulb, rostral migratory stream, brain stem, and hypothalamus/pituitary axis [12,13]. In addition, astrocytes and endothelial cells isolated from the blood–brain barrier also harbored AhR, as its expression is not restricted to neuronal progenitors or neurons [14,15]. AhR is also expressed in glial cells [16], and it is associated with neuronal survival [17]. At the murine suprachiasmatic nucleus, AhR expressions, in terms of AhR mRNA level, also exhibit chrono-periodic and periodic variations [18,19]. The regulation of AhR expression in the nervous system depends not only on internal stimuli, but also on external stimulation such as traumatic brain injury (TBI), strokes, and neuropathic pain [20,21,22,23].

As thrombin plays a crucial role in the development of neurodegenerative disorders, the regulation of thrombin-induced cascade by AhR needs to be determined. In the current study, we used the primary culture of wild type or AhR deleted microglia and an animal model for in vitro and in vivo studies. First, we used the ligand protein docking simulation to assess molecular recognition between the thrombin and AhR. Second, we used primary culture of wild type or AhR deleted microglia and BV-2 cell lines to study the molecular mechanisms. Third, both wild type and AhR knockout mice were injected with thrombin at the hippocampus, and their neurobehavior and immunohistochemistry and associated proteins were assessed. 

## 2. Results

### 2.1. A Novel Docking Site (Ser 36) between Thrombin (Yellow) and AhR (Red) Revealed in the Silico-Prediction of Protein–Protein Molecular Docking Interaction Network 

**Figure 1:**
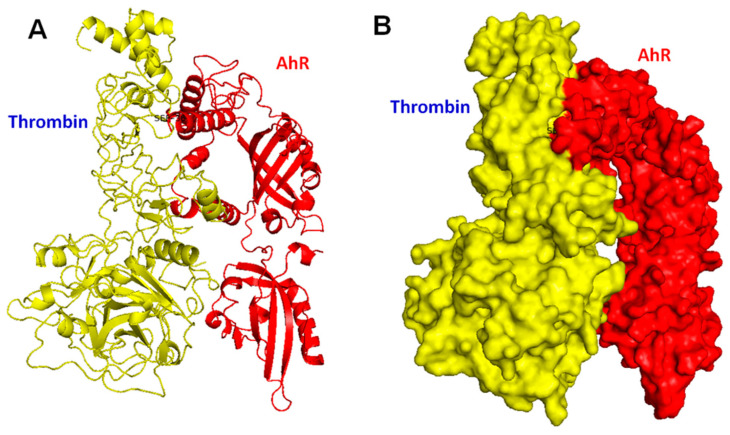
Molecular docking interactions of thrombin at the binding site of AhR (Ser 36) protein. The docking algorithm was tested based on both bound and unbound cases as in the ZDOCK benchmark 2.0 dataset. (**A**) The molecular structure of human AhR is depicted as a ribbon diagram, showing α-helices, β-pleated sheets, and loops. The binding site between thrombin (yellow) and AhR (red) is targeted at Ser 36. The 3D representation of interactions between thrombin and the AhR active site (Ser 36) is generated with the PyMOL(TM) 1.7.4.5.edu. (**B**) Configuration showing the surface representation of the thrombin (yellow) directly targeting AhR (Ser 36). Thrombin (yellow) and AhR (red) are shown in surface representation.

The efficacy of such a method was demonstrated using the ZDOCK benchmark datasets. Results indicated a possible interaction between thrombin and AhR based on structure molecular docking as well as the functional activity of a specific targeting phosphor-ser36 site. The evidence is shown in Figure 1A,B, and it indicates that thrombin is a direct target of AhR (ser36) through post-translational modification and gene regulation.

### 2.2. Aryl Hydrocarbon Receptor (AhR) Deficiency Augmented Thrombin-Induced Microglial NO Release and iNOS Expression and Their Abolishment by AhR Agonist

**Figure 2:**
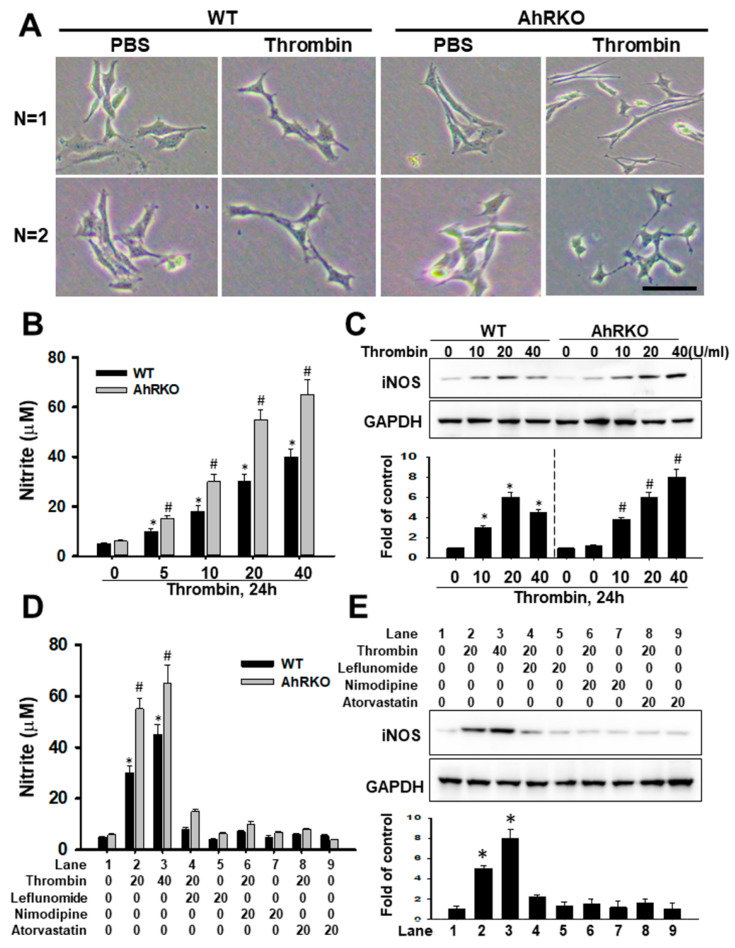
Thrombin-activated microglia cells: release of NO and iNO manipulated by AhR gene knockout and AhR agonist. (**A**) The light photography showed the representative morphology of primary microglia culture either from wild or AhR (−/−) mice subjected to PBS or thrombin stimulation in two respective experiments (N = 1, and 2). (**B**) Microglial cells were incubated for 24 h with the indicated amount of thrombin (U/mL). The amount of nitrite formed from NO was determined as described in Methods. Each value represents the Mean ± SEM of three samples. (**C**) iNO expression was detected by immunoblotting. Cells were treated with 5–40 U/mL of thrombin for the indicated times. Results of quantitative analysis in immunoblotting are shown. (**D**) Microglia were treated with 20 U/mL of thrombin for 14 h in the absence or presence of 20 μM leflunomide (Lef), Nimodipine (Nim), or Atorvastatin (Ator). The amount of nitrite formed from NO was determined. Each value is the mean ± SEM of three samples. *: *p* < 0.05, indicating a significant difference between experimental and control groups; #: *p* < 0.05 indicating a significant difference between wild type and AhRKO groups. (**E**) For immunoblotting analysis, cells were treated with 20–40 U/mL of thrombin for 24 h in the absence or presence of Lef, Nim, or Ator. Results of the quantitative analysis in immunoblotting are shown *: *p* < 0.05 indicated a difference in iNOS expression treated with Lef, Nim, and Ator against treatment with 20 U of thrombin. Bar length = 100 μm.

The nitrite formation and iNOS expression are hallmarks of microglia activation. Primary microglia cells culture obtained from WT and AhR (−/−) mice were first treated with thrombin at 5–20 units/mL for 28 h. The representative morphology of primary microglia culture either from wild or AhR (−/−) mice subjected to PBS or thrombin stimulation are shown in Figure 2A. The photography showed the increased ramification either in wild or AhR (−/−) microglia triggered by thrombin and more ramification seemed to be in AhR (−/−) group. Nitrite production was increased in a dose-dependent manner: 10–40 μm nitrite was detected from 5 × 10^4^ microglia cells treated with either 5, 10, or 20 units/mL of thrombin, compared with 4.7 μm nitrite detected from untreated cells (Figure 2B). However, in primary microglia cells from AhR−/− mice, nitrite production was markedly increased in a dose-dependent manner: 16–65 μm nitrite detected from 5 × 10^4^ cells treated with either 5, 10, or 20 units/mL of thrombin, compared with 6 μm nitrites detected from untreated cells. The iNOS expression was also measured by immunoblot analysis. Results showed a trend of escalating increase for up to 24 h, and expression was maintained steadily for 25 h (Figure 2C). 

We subsequently examined whether an AhR pathway was involved in the thrombin-induced nitrite production. The increasing trend of nitrate production in microglia cells was subjected to thrombin stimulation that presented in escalating doses, and the response was further aggravated with AhR deletion. The administration of an Aryl hydrocarbon receptor agonist, such as Leflunomide, Nimodipine, or Atorvastatin, and nitrite production appeared to counteract this elevation (Figure 2D).Thrombin-induced iNOS expression was also inhibited in the presence of an Aryl hydrocarbon receptor agonist, such as Leflunomide, Nimodipine, or Atorvastatin (Figure 2E). The reduced nitrite production and iNOS expression were not caused by any toxicity of these reagents, as the exclusion of trypan blue was still observed through a light microscope. These data, therefore, strongly suggested that the AhR pathway was involved in the thrombin-induced detrimental effects. 

### 2.3. Aryl Hydrocarbon Receptor Deficiency (AhRKO) Augmented Vascular Permeability In Vitro and Increased Vascular Leakage in Thrombin-Injected Brain Injury Animals

**Figure 3:**
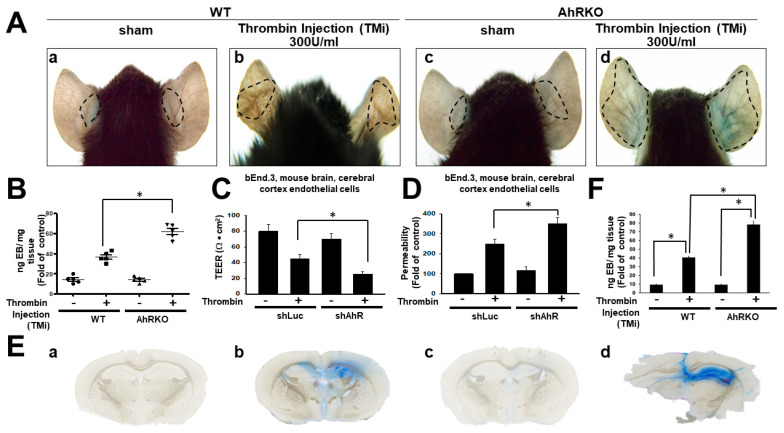
Aryl hydrocarbon receptor deficiency (AhRKO) induced vascular permeability in vitro and increased vascular leakage in thrombin-induced brain injury animals. (**A**) The Miles assay was used to test vascular permeability in vivo by photoimaging vascular leakage in both ears of mice, with subcutaneous regions under various conditions as indicated. (**A**) Leakage of the dye to adjacent tissue was found after treatment with thrombin injection (TMi) 300 U/mL. (**B**) Showing quantified results of vascular permeability, with normalized values relative to the thrombin group and relative changes (Evans’s blue (EB)/mg tissue). Data are presented as mean ± SEM of five independent experiments. * *p* < 0.05 compared with the TMi group. (**C**) Transepithelial/endothelial electrical resistance (TEER) was used to detect barrier tissue integrity in vitro. Endothelial cells of cerebral cortex of mouse (bEND.3) were seeded on transwell inserts for measurement of maximal electric resistance, and were then incubated with thrombin (20 U/mL) until day 4. Transendothelial electrical resistance (TEER, Ω·cm^2^) was monitored using a Millicell-ERS2 Volt-Ohm Meter. (**D**) Cell membrane permeability test. The bEND.3 cells were pretreated with thrombin (20 U/mL). At the end of the treatments, fluorescein isothiocyanate–dextran (FD40, final concentration 1 mg/mL) was added to the cells compartment. Monolayer hyperpermeability was assessed fluorometrically at 485/520 nm, with results showing values normalized relative to the thrombin group and relative arbitrary changes. (**E**) Showing quantified results of vascular permeability, with normalized values relative to the thrombin group and relative changes (Evans’s blue (EB)/mg tissue). Data are presented as mean ± SEM of three independent experiments. (**F**) Leakage of the dye to the hippocampus was found after treatment with thrombin injection (TMi) 300 U/mL either in wild or AhRKO mice. a: sham (wild type); b: thrombin injection (TMi) 300 U/mL either in wild type animal; c: sham (AhRKO); d: thrombin injection (TMi) 300 U/mL either in AhRKO animal. * *p* < 0.05 compared with the TMi group.

The Miles assay is typically used for assessing vascular permeability in vivo by photoimaging the extravasation of injected dye on both ears of mice. Increased leakage of the Evan blue dye to adjacent tissue was shown after treatment with thrombin injection (TMi) at 300 U/mL, and further increased extravasation was observed in AhR (−/−) mice (Figure 3A). The quantitative data revealed that the thrombin-induced two-fold increment in wild type was further increased to three-fold with AhR deletion (Figure 3B). The transepithelial/endothelial electrical resistance (TEER) (Ω·cm^2^) in wild type brain cortex was reduced by adding thrombin, and TEER was further reduced in the cortex of AhR-deleted mice (*p* < 0.05) (Figure 3C). The cell permeability test reciprocally showed an increased permeability in wild type subjected to thrombin stimulation which was further increased in AhR (−/−) animals (*p* < 0.05) (Figure 3D). The quantitative analysis in Evan blue leakage in hippocampus either in wild or AhRKO mice subjected to thrombin injection are shown in Figure 3D. The representative photograph is shown in Figure 3E.

### 2.4. Increased Pro-Inflammatory Cytokine Expressions in Thrombin Treated Primary Microglia Were Augmented by AhR Deletion but Counteracted by Aryl Hydrocarbon Receptor Agonists 

**Figure 4:**
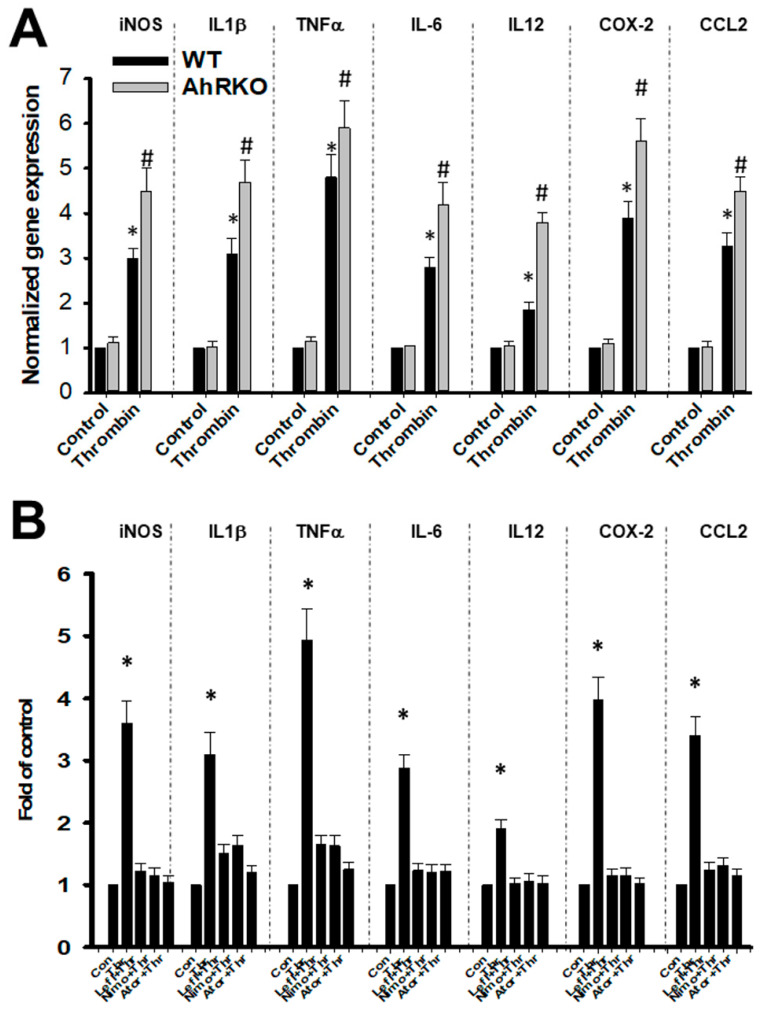
Pro-inflammatory gene expressions in thrombin-triggered microglia were influenced by AhR deletion or AhR agonists. (**A**) Primary microglia cells in either wild type or AhR deleted condition were first treated with thrombin for 12 h, and then their pro-inflammatory cytokines were quantified by qRT-PCR. The pro-inflammatory cytokines studied were iNOS, IL1β, TNF-α, IL-6, IL12, PGE2, and CCL2. AhR deleted microglia showed a significant induction in all pro-inflammatory cytokines’ gene expressions compared with WT microglia. (**B**) These pro-inflammatory gene expressions in thrombin-induced microglia cells were counteracted by AhR agonists. Primary microglia cells were either treated without (control) or with thrombin 20 U/mL for 12 h with the addition of either leflunomide, nimodipine, or atorvastatin, and then their gene expressions (as specified above) were quantified by qRT-PCR. These thrombin-induced cytokine expressions were attenuated by AhR agonists such as leflunomide, nimodipine, or atorvastatin (values shown are mean and SEM; Student’s *t* test; n = 6). *: *p* < 0.05 indicated the statistical significance between the thrombin and control group in wild type microglia cells.; #: *p* < 0.05; indicated the statistical significance between wild and AhRKO group after thrombin treatment.

To determine how inflammatory responses in microglia were influenced by AhR, we measured the gene expression profile of pro-inflammatory cytokines in the wild type or AhR deleted primary microglia culture following thrombin treatments. Gene expressions of iNOS, IL1β, TNF-α, IL-6, IL12, PGE2, and CCL2 were markedly escalated after thrombin treatment in the wild type. With AhR deletion, all the above gene expressions were furthermore escalated (Figure 4A). When the above conditions were given AhR agonists such as leflunomide, nimodipine, or atorvastatin, the thrombin-induced gene expressions were counteracted (Figure 4B). These findings indicated that in microglia, thrombin had triggered inflammatory responses that were modulated by AhR. 

### 2.5. Aryl Hydrocarbon Receptor Deficiency (AhRKO) after Thrombin Injection In Vivo, Increased MMP Activity, but Not MMP2 

**Figure 5:**
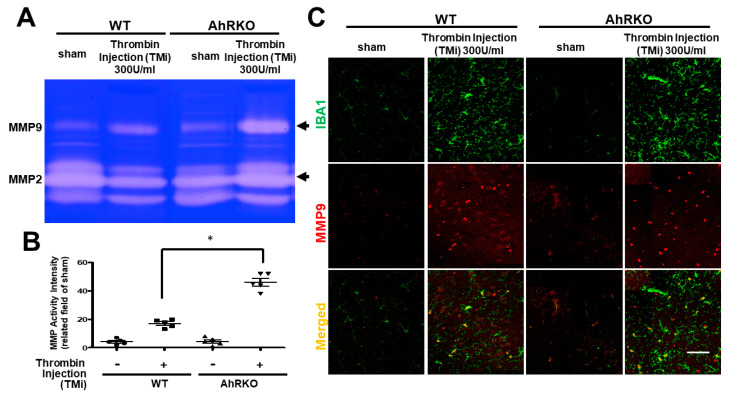
Aryl hydrocarbon receptor deficiency (AhRKO) after thrombin injection in vivo increased MMP9 activity, but not MMP2. AhRKO mice following TMi increased MMP9 activity at 72 h (n = 5, * *p* < 0.05, vs. thrombin vehicle; one-way ANOVA with Fisher’s least significant difference test). (**A**) Presentation of MMP 9 activity. (**B**) Quantification of MMP 9 activity (n = 5, * *p* < 0.05 vs. thrombin group). M2 marker Arginase-1 staining results (n = 5, * *p* < 0.05 vs. thrombin group). (**C**) Representative images of brain sections at 72 h after thrombin injection, showing double staining with both microglia cell marker Iba1 (green) and MMP 9 (red). Scale bars: 50 μm. Data shown are mean ± SEM.

MMP are capable of degrading all kinds of extracellular matrix proteins, but can also process a number of bioactive molecules such as cell proliferation, migration (adhesion/dispersion), differentiation, angiogenesis, apoptosis, and host defense. The increased MMP 9 activity was found in microglia subjected to thrombin induction in wild type but further escalated in AhR deletion (Figure 5A,B). The expression of MMP-9 showed the co-localization with microglia (Figure 5C). The quantitative analysis is shown in Supplementary Figure S1. The analysis also showed the same trend as in the study of zymography.

### 2.6. Aryl Hydrocarbon Receptor Deficiency (AhRKO) after Thrombin Injection In Vivo Revealed an Increase in Pro-Inflammatory M1 Marker and a Decrease in Anti-Inflammatory M2 Marker

**Figure 6:**
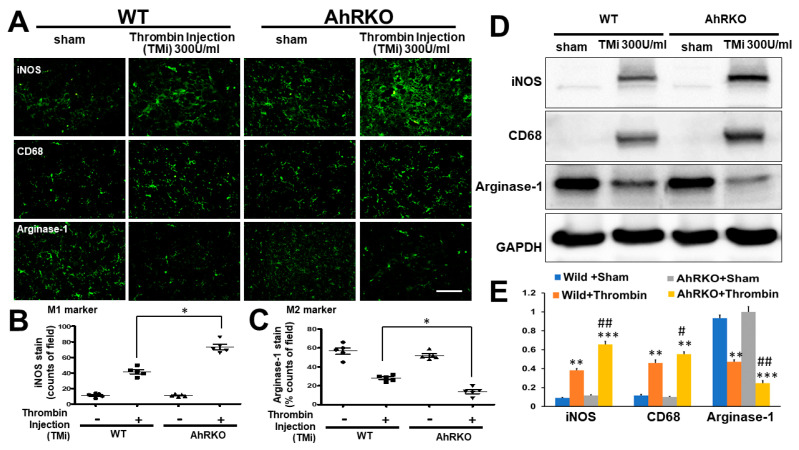
AhR deletion increased the ratio of M1/M2 markers in microglia after thrombin injection in vivo. (**A**) Representative images showing iNOS (M1 marker), microglia marker (IBA-1), and Arginase-1 (M2 marker) in both wild type and AhR-deleted mice three days after thrombin injection (300 U/mL). (**B**) Quantified results of staining with M1 marker iNOSing. (n = 5, * *p* < 0.05 vs. thrombin group). (**C**) Quantified results of staining with M2 marker (Arginase-1) (n = 5, * *p* < 0.05 vs. thrombin group). (**D**) The representative western blot analysis in hippocampus in iNOS, CD 68, and Arginase 1 (GAPDH as an internal control). (**E**) Quantified results of western blot of iNOS, CD 68, and Arginase relative to GAPDH. ** *p* < 0.01 and *** *p* < 0.001 indicated the experimental group relative to sham. # *p* < 0.05 and ## *p* < 0.01 indicated the group of AhRKO relative to wild type after thrombin injection. Scale bars: 50 μm. Data shown are mean ± SEM.

Phenotypes of macrophages are changed by a variety of factors and consequently affect their function. Activated macrophages are typically divided into two categories—M1-like macrophages and M2-like macrophages. M1 macrophages are involved in pro-inflammatory responses and M2 macrophages are involved in anti-inflammatory responses. Thrombin-induced activation of microglia with the predominant M1 cells was revealed by iNOS staining. Deleting AhR further escalated the activation response. Thrombin triggered the downregulation of M2 expression as demonstrated by arginase staining. Deleting AhR, on the other hand, further lowered the M2 expression (Figure 6A). Quantitative results from wild type condition showed a significantly higher expression in M1, and that expression was further escalated by deleting AhR (Figure 6B). For the expression of the M2 marker (Arginase-1), the response was opposite to that of the M1 marker (Figure 6C). In the western blot analysis, the data also showed the same trend as in the result of immunohistochemistry staining in Figure 6D,E. These findings confirmed that thrombin had triggered microglia activation through increasing the M1/M2 ratio, and deletion of AhR further augmented such a response.

### 2.7. Aryl Hydrocarbon Receptor Deficiency (AhRKO) Mice, after Thrombin Injection (TMi), Showed Larger Lesion Volumes and Poorer Neurobehavioral Outcomes

**Figure 7:**
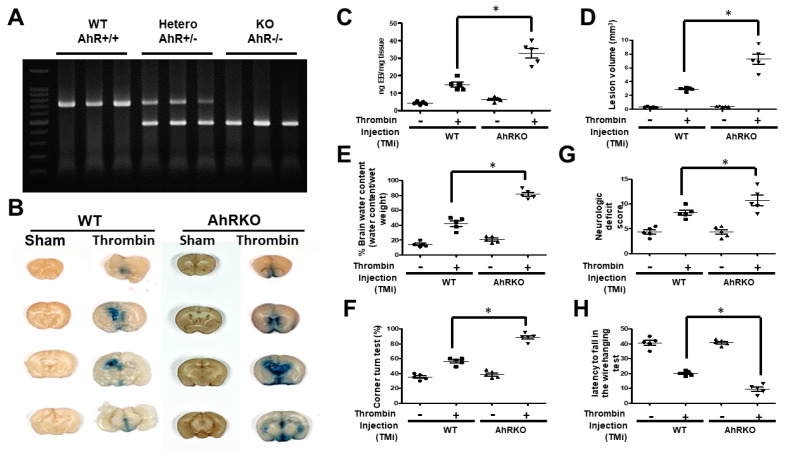
Thrombin injection (TMi) increased lesion volume and impaired neurobehavior, and these responses were further aggravated with aryl hydrocarbon receptor deficiency (AhRKO). (**A**) Results of polymerase chain reaction for AhR genotyping in wild type, AhR +/−, and AhR +/+. (**B**) The representative of photography in Evan blue injection in consecutive of brain slice in coronal view. (**C**) Quantitative data of lesion size measured by the amount of Evan blue in ng. (**D**) Quantified data of lesion volume measured in mm^3^. The volume was calculated by the thickness of a brain slice multiplying the summed surface area of Evan’s blue after thrombin injection derived from contiguous coronal sections. AhR-deleted mice after TMi at 72 h showing the increased lesion volume compared with the wild type (n = 5, * *p* < 0.05, vs. thrombin vehicle; one-way ANOVA with Fisher’s least significant difference test at each time point). (**E**) AhR deletion aggravated striatal edema at 72 h after thrombin injection compared with the wild type (n = 5, * *p* < 0.05 vs. thrombin; Fisher’s least significant difference test). (**F**) AhR deletion impaired corner turn test performance of mice at 72 h after thrombin injection compared with the wild type (n = 5, *p* < 0.05 vs. thrombin; one-way ANOVA with Fisher’s least significant difference test at each time point). (**G**) AhR deletion significantly increased neurologic deficit scores over the wild type at 72 h after thrombin injection. (n = 5, * *p* < 0.05, *t*-test at each time point). (**H**) Wild type mice receiving thrombin injection showed shorter fall latency in the wire hanging test at 72 h after injection, and AhR deletion furthermore led to a longer fall latency. The difference was statistically significant (n = 5, * *p* < 0.05 vs. sham; two-way ANOVA with Fisher’s least significant difference test). Values are mean ± SEM.

The above findings indicated that thrombin had triggered microglia activation, a response that was aggravated by deleting AhR. To further explore the in vivo effect, we stereotactically injected thrombin into the brain at the hippocampus to investigate changes in AhR expression, brain pathology, and neurobehavior. First, to ensure AhR gene deletion, we conducted AhR genotyping by polymerase chain reaction to confirm the deletion success (Figure 7A). The lesion volume was assessed based on the distribution of Evan’s blue stain. We found a significantly larger volume after thrombin injection which was further increased after deleting AhR (Figure 7B,C). To assess brain edema after thrombin injection, we determined the proportion of water in the brain. We found an increased water content in wild type animals after thrombin injection, and that increase was further aggravated with AhR deletion (Figure 7D). We lastly applied the corner turn test, neurological deficit scores, and tail-flick latency study neurobehaviors. In the corner turn test, thrombin injection increased the ratio relative to PBS injection, and AhR deletion further increased this response (Figure 7E). Similar response patterns were found with the neurological deficit score and tail-flick latency (Figure 7F,G). 

### 2.8. Aryl Hydrocarbon Receptor Deficiency (AhRKO) Impaired Neuronal Survival in Organotypic Hippocampus Slice Cultures 

**Figure 8:**
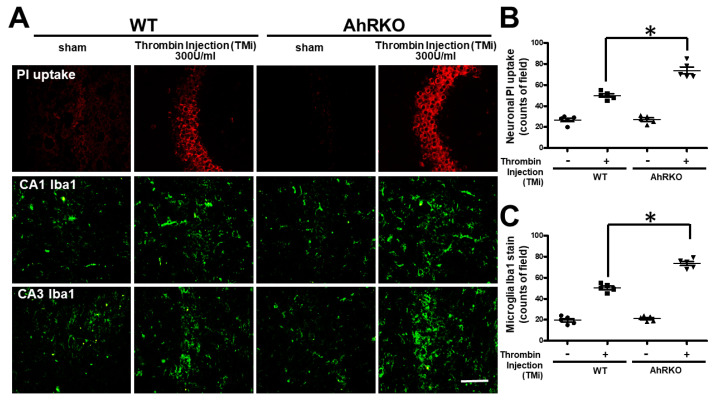
Aggravation of neuronal survival occurred concurrently with activated microglia in organotypic hippocampal slice cultures with hydrocarbon receptor deficiency. (**A**) Neuronal injury at the hippocampal CA1 and CA3 regions is represented by the intensity of PI fluorescence (red), and microglia activation is represented with microglia Iba1 staining labeled with FITC (green). Representative images of hippocampus after TMi injection were allocated to saline (sham) and 300 U/mL thrombin either in the wild type or AhR deleted slice culture. (**B**) Quantified results of neuronal PI fluorescence uptake (count/field). Hippocampal injury was generated by injection of thrombin and observed later at 72 h. AhR deletion aggravated hippocampal injury as induced by Thrombin injection (TMi) 300 U/mL when compared with the wild type at 72 h (n = 5, * *p* < 0.05 vs. Thrombin group; Fisher’s least significant difference test). PI: propidium iodide (red). (**C**) Hippocampal injury is quantified by the intensity of microglia Iba1 staining (count/field) in the fluorescence FITC images of the hippocampal slices, after exposing to thrombin as indicated for 72 h (n = 5, * *p* < 0.05 vs. Thrombin group; Fisher’s least significant difference test). Scale bar: 500 μm. Data are mean ± SEM.

Neurobehavioral impairments are highly correlated with dysfunctional alterations of neurons. To investigate the effects of activated microglia on neurons, we used the hippocampus slice culture approach, injected it with thrombin under a microscope, and then analyzed it three days after injury. Neuronal PI fluorescence uptake measurements showed an increased uptake after thrombin injection that further increased in the AhR-deleted condition. The number of activated microglia was augmented by thrombin, and that further increased after AhR deletion (Figure 8A). Quantitative analyses confirmed the above image findings (Figure 8B,C). A reciprocal relationship was found with AhR deletion in those more active microglia that appeared concurrently, with fewer neurons surviving. 

### 2.9. Aryl Hydrocarbon Receptor Deletion Accelerates Neuronal Death after Thrombin Injection In Vivo

**Figure 9:**
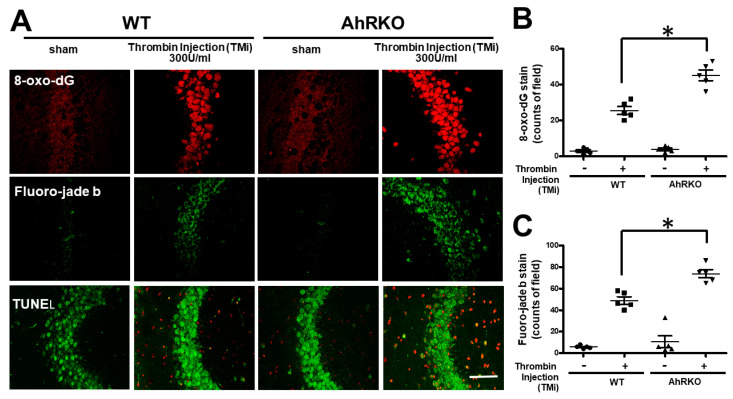
Aryl hydrocarbon receptor deficiency (AhRKO) induced cell death after thrombin injection in vivo. (**A**) Images of brain sections at 72 h after thrombin injection, showing DNA oxidative damages as indicated by 8-oxo-dG staining, neuronal degeneration by Fluoro-Jade B (FJB)-staining, and DNA fragmentation by terminal deoxynucleotidyl transferase dUTP nick end labeling (TUNEL) (red color) and neuron (green color). (**B**) Quantified data on 8-oxo-dG-stained cells (n = 5, * *p* < 0.05 vs. thrombin group). (**C**) Quantified results of Fluoro-Jade B (FJB)-staining (n = 5, * *p* < 0.05 vs. thrombin group). Scale bars: 50 mm. Data are mean ± SEM.

The inflammatory response accelerated in microglia by thrombin or combined effects of activated microglia or thrombin itself may contribute to the neuronal death. To further assess the effects of activated microglia on neurons, either the wild type or AhR-deleted animals were subjected to stereotactic injections of thrombin. Neuronal survival markers such as 8-oxo-dG, Fluoro-Jade B, and TUNEL were used to assess the AhR effects. Representative photo-images images showing the expression of above markers are shown in wild type or AhR deleted animals after thrombin injections (Figure 9A). Quantitative analyses of eight oxo-dG showed that thrombin had triggered 8-oxo-DG expression in the wild type animals, and AhR deletion further exaggerated the response (Figure 9B). Results of Fluoro-Jade B also showed a similar picture (Figure 9C). The TUNEL test also revealed the same phenomenon (Figure 9A). 

### 2.10. Summary of the above Data in Carton Illustration of Hypothesis and a Small Table to Summarize the In Vitro and In Vivo Experiment

To summarize the results in a concise and qualitative way, the graph consists of the hypothesis of AhR involved in thrombin-induced microglia cell activation and the tables of in vitro and in vivo experiment to make this study clearer (Supplementary Figure S2). 

## 3. Discussion

Thrombin is a common detrimental factor that has contributed to neurodegenerative disorders, and AhR is widely distributed in the central nervous system in both neurons and microglia. At present, the modulation of AhR related to the development of thrombin-induced neurodegenerative disorder remains undetermined. In this study, we found that thrombin integrated well with AhR based on ligand protein docking simulations. Thrombin markedly induced the inflammatory response in microglia, and deletion in AhR furthermore augmented the response, increasing the blood–brain barrier permeability, MMP-9 activity, and M1/M2 ratio. This is the first study on the effect of AhR in modulating microglia activity in the context of thrombin-induced inflammatory response at the central nervous system. Results implicated the AhR role in developing thrombin-induced neurodegenerative disorders. 

In the CNS, iNOS is expressed in various glial cells, including astrocytes and microglia, in response to different stimuli [24,25]. However, iNOS is not constantly present in cells, and it is only expressed when the cell is induced or stimulated, typically by proinflammatory cytokines and/or bacterial lipopolysaccharide (LPS) [26,27]. Overexpressing or dysregulating iNOS can result in toxic effects, which is associated with a variety of human diseases, including septic shock, cardiac dysfunction, pain, diabetes, and cancer [26]. The synthesis of NO, a reaction catalyzed by iNOS by activated macrophages, is an important cytotoxic/cytostatic mechanism of non-specific immunity [28,29]. Activated microglia can produce inflammatory mediators such as TNF-α, as well as potentially neurotoxic factors including NO and prostaglandins, which are synthesized by iNOS and COX-2, respectively. NO produced by activated microglia is toxic to neighboring cells [30,31,32]. There were significantly high expressions of iNOS and NO in microglia cells following thrombin treatment both in vitro and in vivo [33,34]. In this study, we found that thrombin treatment had triggered the production of iNOS and NO in the wild type microglia cells. The AhR deletion aggravated the response. AhR agonists produced the opposite reaction. Results showed that AhR played a crucial role in development of the activation of microglia triggered by thrombin. 

BBB functions as a dynamic interface that regulates the brain homeostasis and protects the CNS. BBB responds to different physiological and pathological conditions [35]. Microvascular endothelial cells, the capillary basement membrane (BM), astrocytes, pericytes (PCs), microglial, and neuronal cells form the structure of BBB together [36]. Increased permeability is a well-characterized effect of thrombin and is widely known as a permeability model of endothelial cells (ECs) [37,38]. Thrombin affects cell morphology and intracellular contraction by reorganizing cytoskeleton, and it thus enhances EC paracellular permeability [39] and opens tight junctional complexes [40]. Under inflammatory conditions, the TJs between endothelial cells may be disrupted by cytokines and other pro-inflammatory agents. In addition, mononuclear leukocytes, monocytes, and macrophages can enter the CNS via transcellular and paracellular routes, playing roles complementary to those of the resident microglia. These immune cells may also transform into the microglia phenotype [41]. Several in vitro and in vivo studies showed that BBB is opened by mediators, such as glutamate, aspartate, taurine, ATP, endothelin-1, NO, TNF-α, and macrophage-inflammatory protein 2 (MIP2), which are produced by astrocytes [42,43,44]. Other humoral agents, known to exert similarly opening effect of BBB, are bradykinin, 5HT, histamine, thrombin, UTP, UMP, substance P, quinolinic acid, platelet-activating factor, and free radicals. Their origins vary. Some are released by the endothelium and have an autocrine effect on itself. In this study, thrombin increased vascular permeability and deletion of AhR furthermore aggravated the effect (as demonstrated by the Mile’s assay and Transepithelial/endothelial electrical resistance and transmembrane permeability). In addition to a direct toxic effect from activated microglia on BBB disruption, the pro-inflammatory response and oxidative stress derived from microglia also contributed to the BBB breakdown.

Microglia activation is associated with changes in the expression of cell surface receptors, unique polarization responses, and the release of a variety of inflammatory mediators that contribute to either a tissue reparative role or a neurotoxic response. In general, the classically activated M1 phenotype is associated with pro-inflammatory and neurotoxic responses, while the M2 phenotype mostly mediates anti-inflammatory and neuroprotective functions [45]. M1-like microglia cells upregulate pro-inflammatory cell surface markers, such as MHCII and the cluster of differentiation marker 86 (CD86) [46,47]. They also induced the production of a variety of pro-inflammatory mediators, such as cytokines, tumor necrosis factor-α (TNFα), and interleukins (IL-1β, IL-6, IL-12, IL-17, IL-18, IL-23), chemokines such as CCL12 and CXCL10, and other pro-inflammatory mediators such as reactive oxygen and nitrogen species (ROS and RNS), inducible nitric oxide synthase (iNOS), and cyclooxygenase-2 (COX-2) [47,48,49,50]. M1-like microglia elicit innate immune responses to combat foreign pathogens and trigger the adaptive immune response [51]. Their chronic activation under pathological conditions leads to neuroinflammation, oxidative stress, and neurotoxicity [51,52]. M2 polarized microglia can assume an ‘alternatively activated’ or ‘acquired deactivation’ state, and they are often associated with functions such as immune resolution and tissue repair through the secretion of anti-inflammatory and neurotrophic factors [45,52,53]. M2 microglia are activated by four main anti-inflammatory cytokines—IL-4, IL-10, IL-13, and TGF-β. Both IL-4 and IL-13 promote the alternative activation state and antagonize M1 pro-inflammatory responses, such as the production of TNFα, IL-6, and iNOS [54,55]. Thrombin accumulation in the brain exacerbates edema formation, inflammation, and neurodegeneration, partly due to its activation of microglia [56,57,58]. Several in vivo and in vitro studies reported that thrombin-mediated microglia activation results in the upregulation of M1-associated inflammatory mediators such as iNOS, NO, COX-2, MHCII, and pro-inflammatory cytokines, such as IL-1β, IL-6, and TNFα [34,59,60,61,62]. Modulation of the microglia activation predominantly increases the ratio of M1/M2 as predisposed to severe thrombin-induced injury [60,63]. In this study, thrombin treatment induced the polarization of M1 microglia with concomitant expression of inflammatory cytokines. Our findings are consistent with the previously described literature. Deleting AhR escalated the response toward M1 expression, which implicated that AhR had been involved in regulating the ratio of M1/M2 phenotype in microglia when subjected to triggering by thrombin.

Upregulated MMPs in the CNS likely play several detrimental roles, including the promotion of neuroinflammation, disruption of the blood–brain barrier (BBB) [64,65], demyelination, and damaging axons and neurons (especially MMP-1 and MMP-2) [66]. MMPs also participate in the inflammatory cascade through inflammatory mediators and their receptors [67,68]. Moreover, MMPs may contribute indirectly to expand inflammatory responses and tissue damages by generating antigens through the breakdown of myelin or by converting membrane bound TNF-α to the active myelinotoxic form [69]. The expression of MMPs, produced in microglia at sites of inflammation upon activation (such as LPS and Con A) [70,71], has been reported earlier [72,73,74]. In particular, the secreted MMP-2 and MMP-9 appear to be key modulators [72,74]. MMP-9 is considered to function as a tuner and amplifier of immune functions [73]. MMP-9 helps peripheralizing leukocytes to sites of inflammation in response to chemokines [73], acting as a switch and catalyst at the interplay between the innate and adaptive immune systems. It was also implicated in opening the transform route for immune cells into the neutrophil under various disease conditions, such as multiple sclerosis, stroke, and brain injury [74,75,76,77,78,79,80,81]. In this study, thrombin induced the increased expression of MMP-9 as compared with sham, and that response was further enhanced with AhR-deletion. The phenomena appeared also in parallel with increasing expressions of pro-inflammatory factors such as IL-1, TNF-α, and the BBB breakdown. These results confirmed the involvement of AhR in regulating MMP activity in microglia activated by thrombin.

AhR is widely distributed in the brain, such as in microglia, astrocyte, endothelial cells, and neurons [12,13,14,15]. The modulation of AhR is beneficial for various neurodegenerative disorders through the direct or indirect effects, as described above [20,21,22,23]. Thrombin is detrimental to the central nervous system upon head injury, intracerebral hemorrhage, and brain infarction [4,6,8]. In this study, we found that thrombin had exaggerated the harmful response through the involvement of blood–brain breakdown, polarization of microglia, increased oxidative stress, and secretion of inflammatory cytokines, as well as the modulation of MMP activity. The deletion of AhR further augmented such responses, indicating that AhR was involved in modulating thrombin-induced microglia activation. With further clarification of the underlying mechanisms, thrombin-associated brain injuries should be better treated. 

## 4. Materials and Methods

### 4.1. Molecular Docking Modeling 

PyMOL is a widely popular macromolecular visualization system that uses the OpenGL Extension Wrangler Library (GLEW) and Free OpenGL Utility Toolkit (Freeglut). PyMOL uses cross-platform widget toolkit (Tk) for the GUI widgets and can produce high-quality movies and images of macromolecules in different representations such as ribbon, cartoon, dot, surface, sphere, stick, and line. PyMOL can also extend to protein–ligand modeling, molecular simulations (MS), and virtual screening (VS) unities in PyMOL. The computational drug discovery function of PyMOL has been successfully applied to find new drug candidates for various targets [82].

The description of docking simulations with ZDOCK is briefly described below. First, in the protein structure preparation, the three-dimensional structure of AHR (UniProt ID: P35869) and thrombin (UniProt ID: P25116) in the Protein Data Bank (PDB) format were retrieved and prepared for docking. This procedure became involved in checking for any missing residues, adding hydrogen atoms, and addressing any structural irregularities or inconsistencies. Second, in ZDOCK input preparation, the retrieved structures of AHR and thrombin, including the specific ligand position at Ser-36 in AHR, were used as input for ZDOCK. The PDB files of the two proteins information were transferred to the software. Third, in receptor–ligand definition, the ligand position at Ser-36 in AHR was defined as the binding site for thrombin. Fourth, in grid generation, ZDOCK employed a grid-based approach to facilitate the initial search process. A three-dimensional grid was generated around the receptor protein of AHR, focusing on the ligand position at Ser-36. The grid size and resolution parameters were determined based on the characteristics and proximity of the ligand site to the binding partner. Fifth, in Fast Fourier Transform (FFT) search, ZDOCK utilized a fast Fourier transform algorithm to efficiently explore the six-dimensionally translational and rotational space of AHR and thrombin, with specific emphasis on the ligand position at Ser-36 in AHR. Sixth, in Monte Carlo refinement, ZDOCK performed a refinement step using a Monte Carlo algorithm. This step aimed to improve the accuracy of the predicted docking models by adjusting the protein–protein interface and exploring potential conformational changes while maintaining the specific ligand position at Ser-36 in AHR. Seventh, in Scoring Function- ZRANK: ZDOCK employed a scoring function called ZRANK to evaluate and rank the predicted docking models. ZRANK incorporated various energy terms, such as electrostatic interactions, van der Waals forces, and desolvation energy, to assess the quality of each model. This model was ranked based on their ZRANK scores, with lower scores indicating a more favorable binding model. Finally, in Output Analysis, ZDOCK generated an output file containing a ranked list in the predicted docking models between AHR and thrombin. Each model was assigned a ZRANK score, allowing for the identification of the most favorable docking configurations that accommodated the ligand position at Ser-36 in AHR, which was used in this experiment.

### 4.2. Cell Culture

We obtained C57BL/6 mice (4–5 weeks old, 20–22 g) from the National Applied Research Laboratories (NAR labs, Taipei, Taiwan), and AhR-knockout mice (B6.129Ahrtm1Bra/J) (6–8 weeks old, 18–22 g) from the Jackson laboratory (Bar Harbor, ME, USA). Our method for generating Aryl hydrocarbon receptor (AhR) deficiency has already been published [21,83]. The microglia were first harvested from both wild-type and AhR (−/−) C57BL/6J mice for the following study. The method of microglia culture was described previously [84,85]. In brief, cortical cell cultures of mice were prepared from the 17-day-old fetal brains, and neocortices were mechanically triturated. Dissociated cells were plated on 12 mm round plastic coverslips in 24-well plates at a density of 1.5 × 10^5^ cells/coverslip. Plating media consisted of Eagle’s minimal essential medium (MEM, Earle’s salts, supplied glutamine-free) supplemented with 5% horse serum, 5% fetal bovine serum (FBS), 21 mM glucose, 26.5 mM bicarbonate, and 2 mM L-glutamine. Cortices were triturated into single cells in DMEM containing 10% fetal bovine serum and plated into 75 cm^2^ T-flasks (0.5 hemispheres per flask) for 2 weeks. Microglia were detached from the flasks by mild shaking and applied to a nylon mesh to remove astrocytes and cell clumps. Cells were plated into 24-well plates. Plates were washed 1 h later with medium to remove unattached cells. In some experiments, immortalized microglial cells of cell line BV-2 were cultured and maintained in DMEM medium containing 10% heat-inactivated low endotoxin FBS (Life Technologies, Carlsbad, CA, USA) and streptomycin/penicillin (Life Technologies) in a humidified atmosphere containing 5% CO_2_. 

### 4.3. Stereotaxic Surgery and Drug Injection

Either the wild-type or the AhR (−/−) C57BL/6J mice (25–30 g) were first anesthetized with an injection of chloral hydrate (400 mg/kg i.p.) before being positioned in a stereotaxic apparatus. Thrombin solution (20 U) was stereotactically injected into the right cerebral cortex (AP + 1.4 mm ML, −2.0 mm, DV −2.0 mm from bregma) [84,85]. In this study, 20 U of thrombin dissolved in 5 μL of phosphate-buffered saline were injected at a rate of 0.5 μL/min through a 26-gauge Hamilton syringe needle driven by an automated pump. In the hippocampus slice culture, 20 U of thrombin dissolved in 5 μL of phosphate-buffered saline was also slowly injected through a 26-gauge Hamilton syringe needle driven by an automated pump. 

### 4.4. Immunohistochemistry

Animals were transcardially perfused with a saline solution containing 0.5% sodium nitrate and heparin (10 U/mL) followed by 4% paraformaldehyde dissolved in 0.1 M phosphate buffer (PB). Tissues obtained from either the animal cranium or hippocampus slice were postfixed for 1 h, washed in 0.1 M PB, and then immersed in 30% sucrose solution until the tissues had sunk. Tissues were sectioned on a sliding microtome at a thickness of 40 μm, and every sixth serial section was selected and processed for immunostaining as described previously [84]. In brief, brain sections were incubated in 0.2% Triton X-100 for 30 min, rinsed twice in PBS with 0.5% bovine serum albumin (BSA), and then incubated overnight at room temperature with the appropriate primary antibodies. The primary antibodies used were against one of the following: iNOS (1:300, Merck Millipore, Burlington, MA, USA), MMP2 (1:500, Abcam, Cambridge, UK), MMP9 (1:500, Santa Cruz, Heidelberg, Germany), Arginase (1:200, Santa Cruz), 8-oxo-DG (1:500, Merck Millipore), Furo-jade (1:200, Merck Millipore), IBA1 (1:400; Serotec, Kidlington, UK), and NeuN (1:500; Chemicon International, Temecula, CA, USA). Sections were rinsed thrice and subsequently incubated with FITC-labeled anti-mouse IgG (1:200; Kirkegaard and Perry, Gaithersburg, MD, USA) and Texas red-labeled anti-rabbit IgG (1:200; Vector, Stuttgart, Germany), or with Texas Red-labeled anti-mouse IgG (1:200; Vector) and FITC labeled anti-goat IgG (1:200; Organon Teknika, Durham, NC, USA) for 1 h at room temperature. Tissues were finally washed and glass-mounted with a Vectashield mounting medium (Vector). Stained cells in glass slides were viewed using an Olympus IX71 confocal laser scanning microscope (CLSM; Olympus, Shinjuku, Japan). 

### 4.5. Western Blot Analysis

Protein expressions were determined by Western blotting, as described previously [86]. In brief, proteins (60 μg) were separated by SDS-PAGE, electrophoretically transferred to nitrocellulose membranes, and blocked for 1 h in phosphate-buffered saline containing Tween 20 (0.1%) and non-fat milk (5%). Blots were incubated with iNOS (1:200, Merck Millipore) and β actin (1: 200, Santa Cruz) for 1 h. Membranes were then incubated for 1 h with the horseradish peroxidase-conjugated secondary antibody. After further washing with phosphate-buffered saline, blots were incubated with commercial chemiluminescence reagents (Amersham Biosciences, Amersham, UK).

### 4.6. Nitrite/Nitrate Assay

Concentrations of nitrite in the supernatant of cell cultures were determined using the nitrite/nitrate colorimetric assay kit (R & D Systems, Minneapolis, MN, USA). Nitrite, an end-product of NO oxidation, was used as an indicator of NO production. Nitrite in the conditioned medium was determined using the Griess reagent. Absorbency was determined at 550 nm using a thermos-microplate reader (Molecular Devices, San Jose, CA, USA) [86].

### 4.7. Mile’s Assay 

To determine the vascular permeability in mice, Mile’s assay was performed [87]. Experimental animals were first anesthetized, shaved, and intravenously injected with Evans Blue dye (45 mg/kg in 100 μL). Mice were observed for the next 4 h before their skins and ears were photographed, and finally dissected. PBS served as the negative control. The dye was then eluted from the dissected samples with 500 μL of formamide at 55 °C water bath or heat block and incubated for 48 to 72 h to extract Evans Blue from the tissue before measuring optical density at 620 nm with a spectrophotometer (Biotrak II, Amersham Biosciences).

### 4.8. Transepithelial/Transendothelial Electrical Resistance Measurement

The transepithelial electrical resistance was measured with the Millicell-ERS2 Volt-Ohm Meter, and the resistance value (in Ω) for each well was recorded [87]. Before measurements, electrodes were cleaned, equilibrated, and sterilized according to the manufacturer’s instructions. The resistance of a blank well insert (culture without cells) was also measured. To obtain the sample resistance, the blank value was subtracted from the total resistance of the sample. The final unit area resistance (Ω*cm^2^) was calculated by multiplying the sample resistance readings by the effective area of the membrane (0.33 cm^2^ for 24-well Millicell inserts).

### 4.9. Membrane Permeabilization Assay

The method had been described before [87]. After fixation, 8 × 10^4^ cells were harvested at 180× *g* for 10 min, washed once with 20 mL of Tris Buffer Saline (TBS) (50 mM Tris, 150 mM NaCl, pH 7.6), and suspended to a final density of 2.5 × 10^6^ cells/mL in TBS. Similarly, 5 × 10^9^ *E. coli* cells, were harvested at 300× *g* for 10 min, washed once with 20 mL of TBS and suspended to a final density of 2.5 × 10^7^ cells/mL in TBS. Cell suspensions (500 μL each) were aliquoted into 1.5 mL tubes and treated with a permeabilization agent. Permeabilization agents used were: Triton X-100 (0.1% *v*/*v*), Tween-20 (0.2% *v*/*v*), Saponin (from Quillaia bark) (0.1% *w*/*v*) (S4521), and Digitonin (D141) (0.5% *w*/*v*). All agents were from Sigma-Aldrich (St. Louis, MO, USA). Concentrations used were derived from several protocols [87]. Cells were permeabilized for 25 min at 25 °C and shaken at 500 rpm. Permeabilized cells were washed once with TBS (centrifugation speeds as above) and blocked on ice with TBS + 1% *w*/*v* Bovine Serum Albumin (BSA) for 30 min. Blocked cells were exposed to 0.75 μg of Cyanine-5 (Cy5) or Phycoerythrin (PE) labeled Streptavidin (SAv-Cy5, MW = 60 KDa or SAv-PE, MW = 360 KDa) (Biolegend, CA, USA) for 30 min at 25 °C and shaken at 280 rpm. Cells were washed with 1 mL of 0.15 M NaCl solution and resuspended in 350 μL of the same solution for analysis. Bacterial cells were also labeled with 1 μL of Syto^®^ BC (Invitrogen, Waltham, MA, USA) for 5 min and analyzed with a flow cytometer in BD LSRII. We identified four T1 cells and gated them based on their Forward/Side scatter. *E. coli* cells were detected using the 488–1 (Fluorescein isothiocyanate-FITC), 525/50 filter for Syto^®^ BC and gated using the side scatter. Cy5 positive cells were detected with the red 670/14 filter. PE positive cells were detected with the yellow/green 780/60 filter. For each experimental replicate, 3 × 10,000 events were recorded for 4 T1 cells and 3 × 100,000 were recorded for bacteria.

### 4.10. Assessing Lesion Volume with Injected Evan Blue Dye

The lesion volume was estimated based on Evans blue distributed areas in the brain. Briefly, Evans blue (4%, 1 mL/kg) was injected 24 h after reperfusion via the tail vein. Three hours after injection, animals were perfused with heparinized saline solution. The areas of lesion were measured using a computer image analysis system. The volume was estimated based on the summed area multiplied by slice thickness (Alpha Innotech Corporation (San Leandro, CA, USA), IS1000) [88].

### 4.11. Water Content in the Brain

The brain samples were dried in an oven at 110 °C for 24 h, and the water content of these samples was then calculated based on the wet and dry weights as follows: water content (%) = [wet weight − dry weight]/wet weight × 100 [88].

### 4.12. RNA Isolation and Quantitative PCR 

To determine levels of gene expression, total RNA was extracted from either primary microglia, BV2 cells, or brain cortical tissue using the Trizol reagent (ThermoFisher Scientific, Waltham, MA, USA). The isolated and purified RNA was reverse-transcribed into cDNA using a cDNA synthesis kit according to standard protocols. Quantitative PCR (qPCR) was conducted using synthetic primers and SYBR Green on a Bio-Rad iQ5 Multicolor Real-Time PCR Detection System (Hercules, CA, USA). After incubation at 50 °C for 2 min and 95 °C for 10 min, samples were subjected to 35 temperature cycles, each consisting of 15 s at 95 °C and 1 min at 60 °C. Primers for qPCR are listed in Supplementary Table S1. Final results were all normalized as fold-change of the target gene relative to GAPDH.

### 4.13. AhR Genotyping as Obtained by Genomic Polymerase Chain Reaction

Experimental AhRKO mice from the Jackson Laboratory (Strain Name: B6.129-Ahrtm1Bra/J and Stock No. 002831) were bred, and AhR+/+ and AhR−/− C57BL/6 mice were obtained by breeding AhR+/−. Genotyping was conducted by quantitative PCR with primers consisting of the sense of 5′-TTCTATGCTTCCTCCACTATCCA-3′ and antisense of 5′-GGCTTCGTCCACTCCTTGT-3′, according to published procedures [83]. 

### 4.14. Gelatin Zymography 

Gelatinases in cell culture supernatants from wild-type or AhR-deleted mice after exposure to thrombin were determined by sodium dodecyl sulfate-polyacrylamide gel electrophoresis (SDS-PAGE) zymography according to a modified method described before [83]. In brief, 50 µL of culture supernatant was supplemented with 30 µL of electrophoresis-loading buffer containing SDS. Samples were then separated in a 7.5% polyacrylamide gel which had been copolymerized with 0.1% (*w*/*v*) gelatin type B. Stacking gels contained 5.4% polyacrylamide. The electrophoresis was performed at 4 °C for ∼18 h at 80 V. After electrophoresis, gels were washed for 2 × 20 min in 2.5% (*w*/*v*) Triton X-100/10 mM CaCl_2_ in 50 mM Tris-HCl, at pH 7.4 (washing buffer) in order to remove SDS, and then samples were incubated for 24 h at 37 °C in 1% (*w*/*v*) Triton X-100/50 mM Tris-HCl/10 mM CaCl_2_, at pH 7.4 (developing buffer). For the development of the enzyme activity, gels were stained with Coomassie Brilliant Blue R-250 and destained in methanol/acetic acid/water. Gelatinase activity was detected as a white band on a blue background in the image and quantified by computer image analysis using a two-dimensional scanning densitometer with the Image Master 1D program (Pharmacia Biotech, Uppsala, Sweden). Gelatinase activity was expressed as optical density (OD)×mm^2^, representing the scanned area under the curve, taking into account both brightness and width of the substrate lysis zone.

### 4.15. Terminal Dexonucleotidyl Transferase-Mediated Biotinylated UTP Nick-End Labeling (TUNEL) Assay

Serial sections of the brain that were 8 μm thick were cut on a cryostat and mounted on superfrost/plus slides (Menzel-Glaser, Braunschweig, Germany). The TUNEL assay (Roche Molecular Biochemicals, Mannheim, Germany) was carried out as previously described [89]. Apoptotic cells were defined as those containing TUNEL-positive nuclei with features of condensed and fragmented, as assayed by DAPI (Molecular Probes, Eugene, OR; 1:2000 dilutions). The number of apoptotic cells was expressed as a percentage of the total number of nuclei counted (≥25,000 nuclei for each condition) (n = 6 for each group).

### 4.16. Corner Turn Test

The Corner turn test was originally designed to evaluate sensorimotor deficiency in experimental stroke models [90]. It assesses vibrissae sensory impairments and abnormal limb usage [91]. In brief, two boards were attached together at an angle of 30°, with a small opening between them, and animals were encouraged to walk and explore the corner of the boards. The turning of the animals either to the left or to the right side was evaluated for 10 trials (for a valid trial the rodent had to rear), and the laterality index was calculated. Three days before surgery, to normalize the data, a baseline measure was taken first. 

### 4.17. Modified Neurologic Severity Score (mNSS)

The modified neurologic severity score (mNSS) is a widely used test for the neurological evaluations on multiple aspects, including motor function, sensory function, and reflex function. It has a maximum score of 14 [92]. Scores from 1 to 4 indicate mild defects, from 5 to 9 indicate moderate defects, and from 10 to 14 indicate severe defects. 

### 4.18. Latency to Tail-Flick

The tail flick test is a test of the pain response in animals, similar to the hot plate test. It is used in pain research and to measure the effectiveness of analgesics by the reaction time to heat stimulus (in from 46 to 55 °C) [93]. In brief, the mouse is anesthesia-free, and the animal is held fixed by the experimenter through a linen glove, with its tail resting on the groove of the heat panel. After the mouse has relaxed, an infrared radiant heat source is applied onto the tail and the latency of tail flick is recorded.

### 4.19. Hippocampus Slice Culture

The method for hippocampus slice culture was previously described [94]. The head of the animal was cut off with a pair of sharp scissors. The brain was scooped out quickly with a round spoon micro-spatula and placed in the slurry of dissecting solution to chill for ~1 min. Ice-cold dissecting solution of 10 mL was poured onto a 60 mm dish. The brain was transferred from the beaker to the dish to be completely submerged in dissecting solution. Under a dissecting microscope, dissecting tools were used to separate the hemispheres exposing the midbrain, and then hippocampus on both sides. A dissecting needle was used to isolate and clean the entire hippocampus carefully. Using the snipped tip of a P1000 filter pipette tip, the hippocampus was gently aspirated and transferred to the Teflon sheet on the tissue slicer, with the hippocampus placed on its concave side. The hippocampus was aligned perpendicular to the slicer blade to produce coronal sections and to drain the excess liquid. The hippocampus was slice-cut at 400 μm, and 10 mL cold SCM was poured onto a 60 mm dish for receiving hippocampal slices, again using another snipped P1000 filter tip (Corning Life Sciences (Wujiang), Jiangsun, China) and cold SCM. The 6-well plates with SCM and cell culture inserts were removed from the incubator. Together with another snipped P1000 filter tip, four to five individual slices were transferred onto the membrane. Excess medium was removed without touching slices. Plates were placed back into the incubator and cultured at 35 °C and 5% CO_2_ for the assessment of thrombin. 

### 4.20. Staining and Imaging Organotypic Hippocampal Slice Cultures

Two hours prior to fixation with 4% paraformaldehyde (Sigma-Aldrich), PI (5 μg/mL, Merck Millipore) was added to the culture medium. OHSC was removed from the cell culture inserts and washed with phosphate buffered saline (PBS) containing 0.03% (*v*/*v*) Triton X-100 (Applichem, Darmstadt, Germany; PBS-T) for 10 min, followed by an Aqua test for 5 min, and was mounted with DAKO fluorescent mounting medium (DAKO Diagnostika GmbH, Hamburg, Germany). Further analyses were performed with a CLSM (LSM 710 Meta, Zeiss, Oberkochen, Germany). To detect PI-labeled degenerating neurons, monochromatic light with wavelength at 543 nm at an emission band pass-filter from 585 to 615 nm was used. The dentate gyrus of the hippocampus was visualized with a 20× objective to form a z-stack at step-intervals of 2 μm, forming images of 1024 × 1024 pixel at 0.52 μm/pixel [95].

### 4.21. Statistical Analyses

Values in this study were presented as mean ± S.E.M. All analyses were performed by ANOVA followed by a Fisher’s least significance test. Statistical significance was set at *p* < 0.05.

## 5. Conclusions

Ligand protein docking simulations showed a strong integration between thrombin and AhR. Thrombin caused the activation of microglia through increased oxidative stress, increased vascular permeability, greater inflammatory responses, more M1 phenotype, and greater MMP-9 activity. Deleting AhR further augmented the above detrimental effects, thereby indicating that AhR modulation is essential for the regulation of thrombin-induced brain damage.

## Data Availability

Not applicable.

## Supplementary Materials

The following supporting information can be downloaded at: https://www.mdpi.com/article/10.3390/ijms241411416/s1.
