# Supplementary material for: Thrombin-Induced Microglia Activation Modulated through Aryl Hydrocarbon Receptors
Source: Int J Mol Sci. 2023 Jul 13;24(14):11416. doi: 10.3390/ijms241411416 (PMC10380349; doi:10.3390/ijms241411416)
Supplement: Supplementary file 1 [file ijms-24-11416-s001.zip › ijms-2361154-supplementary.pdf]

Supplementary data:

Supplementary Figure S1:

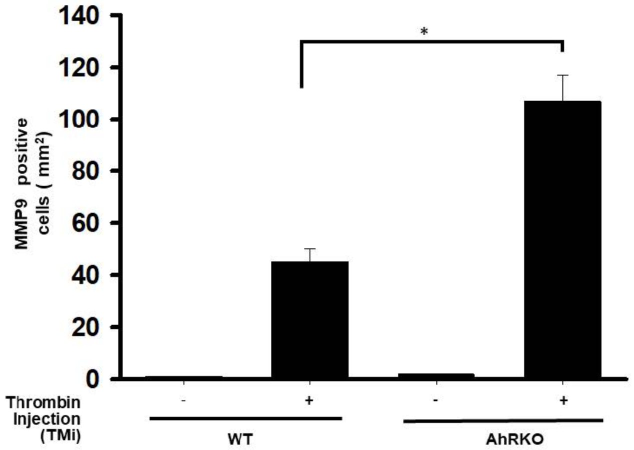

Supplementary Figure S1: The quantitative analysis in number of MMP-9 positive cells by counting the number of double staining with both Iba1 (green) and MMP 9 in the brain sections at 72 h after thrombin injection. The data was presented as number/surface area (mm^2) (n=3). *:$p<0.05$

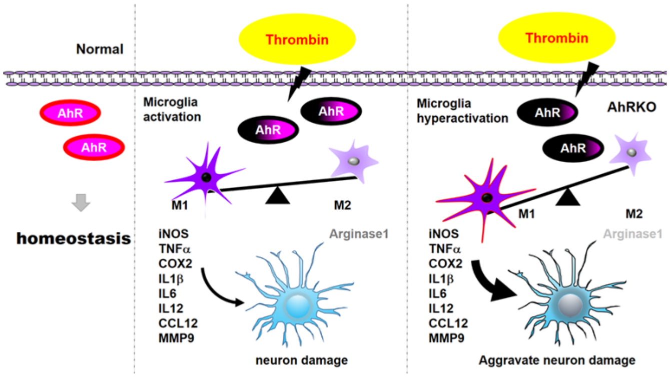

Detection	*In vivo*	Primary microglia *In vitro*
Structure molecular docking		v
iNOS, NO production		v
Cytokine expression		v
Mile assay, TEER, Permeability	v	v
M1/M2 marker expression	v	
MMP activity	v	
Neuron IF stain	v	
Microglia IF stain	v	
Behavior	v	
Functional detection	v	

Supplementary Figure S2: The graph consisting of hypothesis of AhR involved in thrombin induced microglia cell activation and the tables of in-vitro and in-vivo experiment.

Supplementary Table S1: Sequence of primer (mouse) used for RT-PCR

Primer	Forward (5′-3′)	Reverse (5′-3′)
iNOS	GACATTACGACCCCTCCCAC	GCACATGCAAGGAAGGGAAC
IL-1β	TGCCACCTTTTGACAGTGATG	AAGGTCCACGGGAAAGACAC
TNF-α	TGATCGGTCCCCAAAGGGAT	TGTCTTTGAGATCCATGCCGT
IL-6	CAACGATGATGCACTTGCAGA	GTGACTCCAGCTTATCTCTTGGT
β-actin	TGCTGTCCCTGTATGCCTCTG	TGATGTCACGCACGATTTCC
IL-12	AGCACGGCAGCAGAATAAA	CTCCACCTGTGAGTTCTTCAAA
PGE2	GCGACATACTCAAGCAGGAGCA	AGTGGTAACCGCTCAGGTGTTG
CCL2	GCTACAAGAGGATCACCAGCAG	GTCTGGACCCATTCCTTCTTGG
